# The History of Asiatic Cholera

**Published:** 1873-08

**Authors:** H. A. Buck

**Affiliations:** St. Louis


### The History of Asiatic Cholera.

By H. A. Buck, M. D., St. Louis.

"We have good authority for believing that epidemic cholera has its origin in the *Black Death*, or *Oriental plague* of the 14th century. And cholera, unquestionably, in all its various forms has destroyed more lives than all of the other epidemics taken together. We are informed by historians that it swept off one-fourth of the inhabitants of the Old World, in the short period of four years. England lost one-half of its population—London lost 100,000 in four months and other cities in proportion. It was preceded by great revolutions in the earth. From China to the shores of the Atlantic, the foundations of the earth were shaken, the atmosphere was in continual commotion: and endangered by its poisonous influences, both vegetable and animal life. These terrible convulsions are said to have began in 1333, fifteen years before the *plague* broke out in China. According to the traditions of the Chinese, the pestilence was followed by torrents of rain, and great floods, and more than 400,000 people perished in consequence of the floods. A few months afterwards an earthquake caused an extensive range of mountains to sink and disappear, and in its place a lake was formed, more than 100 leagues in circumference—where thousands found a watery grave. And as the water subsided, the flooded districts were drained, foul vapors arose everywhere, from decomposing vegetable and animal matter, made more horrible and poisonous by the odor of putrified corpses, the victims of floods, famines and earthquakes.

It is probably that the atmosphere thus contaminated gave origin to the Black Death, whenever the organs of respiration came in contact with it. At a later period it was stated that its origin in several instances was traced to the pilgrims, who visited the banks of the Ganges, in the observance of their religious rites.

And still later, the havoc committed by the epidemic of Alexandria, suggested official inquiries, and the President of the Board of Health addressed a communication on the subject to the Minister of Foreign Affairs. In this paper it is stated as the opinion not only of the President himself, but of all the scientific and professional authorities in Egypt, that the poison was generated by the crowds of pilgrims periodically visiting the holy places of Arabia. The pilgrims congregate at certain periods of the year, from all parts of the Mohamedan World, to the number of seven or eight hundred thousand.

It is ever a point of religion with them that no Pilgrim should change his clothes during the whole time of his pilgrimage. Under these conditions they are huddled together in enormous crowds beneath the fiery sky of the desert. It is an indispensable incident of the ritual that each pilgrim should sacrifice at least one sheep; the skin and offal of these countless victims are left to decompose under an Arabian sun. The result of all this is that thousands of pilgrims perish on the spot, leaving their bodies to be shuffled hastily under a coating of sand which the first sirocco will disperse; and their clothes to be packed up and carried off as relics to be distributed among their relations and countrymen. The Egyptian minister thinks that here is the seed-plot and hot-bed of cholera.

It is estimated that 4,000,000 people perished in China in the year 1337. Europe was also visited with earthquakes and other atmospheric phenomena. These convulsions continued till 1347, when the plague or cholera, in its primitive form, broke out in the East.

The epidemic entered the Western countries of Asia from China, in the year 1347, and here the historian obtains the first certain knowledge of the character of the disease. From China the route of commerce ran—from thence to Constantinople, the medium of communication between Asia, Europe and Africa. Thus in all directions contagion made its way, and doubtless Constantinople and the harbors of Asia Minor were the great centers of infection, whence it spread all over the world. People were struck down by "*Black Death*" as if by lightning, and the young and strong were more frequently its victims than the aged and infirm. The discriptions of the disease given by historians are rather indifferent They inform us that sometimes it commenced with bleeding at the nose, which was a sure sign of death. Both in men and women, tumors in the groin, and inside of the thigh appeared at the beginning. These varied in size, but were frequently as large as an egg. Similar tumors appeared afterwards all over the body, and black and blue spots came out on the arms and thighs. These spots were indications of the fatal termination of the disease. No power of medicine brought relief; almost all died in from one to three days, and generally without fever and other symptoms—such was the form that the "*Black Death*" assumed in 1347, and three years following. In 1360 and 1373, it assumed a milder form, and then it was called the "*Oriental plague.*" The tumors no longer appeared, and bleeding at the nose was seldom known to occur. It has been supposed by excellent medical authority, that the form thus assumed by the "*Oriental plague,*" is identical with the cholera of the present age, and that the term of *Black Death* applied to it, was derived from the livid, and frequently spotted appearance of patients in cases of malignant cholera. The most reliable estimates given, state that China lost 13,000,000 by *Black Death or Oriental Plague.* India was almost depopulated, and Tartary and Syria, and all the adjoining countries were literary covered with dead bodies. Cyprus, it is said, lost all its inhabitants; and ships without crews were seen, long after, floating about in the Mediterranean. It was reported that in the East, excepting China, 23,000,000 fell victims to this pestilence. No climate was a barrier to its devastating and death dealing power; it penetrated the icy regions of Greenland, Iceland, Norway and Denmark.

It reached the British Isles in 1349, and Russia in 1351, and according to all accounts it was not as fatal in the cold climates as it was in the hot climates. Thus we have the form and character of what is supposed to have been epidemic cholera in its primitive state. Quite a period of time elapses before we have any description of this epidemic again, although it is supposed by some to have prevailed in a milder form in these countries where it first made its appearance. It visited London again in the year 1666, and the mortality averaged one-fourth part of the population. During the cholera of 1832, the worse plague that has visited London since 1666, the deaths were only one out of two hundred and fifty inhabitants. This decrease of mortality was owing to the sanitary improvements in regard to cleanliness.

During the latter part of the 17th century, and until the year 1774, the cholera appeared to have confined its ravages almost exclusively to the Hindoos.

Hindoo practitioners appear to have been familiar with its symptoms, or at least a disease closely resembling it, which they called *vishuchi*, a term in their language signifying vomiting and purging. In the first campaigns of the British troops in India, in the year 1774, cholera, presenting all the symptoms known to characterize the epidemic, made its appearance at Madras, and proved very fatal to both the European and native soldiers, carrying them off very quickly after they were attacked. It is asserted that over 60,000 people perished between the years 1774 and 1781. The disease prevailed at various times between the years 1783 and 1790, and always with the same symptoms and with the same fatal results.

In the year 1783, the cholera broke out at the sacred bathing place of the Ganges. This was the year of the great pilgrimage; it is said 2,000,000 were assembled at this time. In eight days 20,000 fell victims. It did not extend beyond the place of bathing, and ceased on the dispersion of the multitude. In 1817, it broke out again on the banks of the Ganges, causing a greater destruction of life than at any other time. By the end of 1818 its ravages embraced nearly all of Hindostan, and in 1819 it appeared in Java, in the Isles of France and Bourbon, and over India and China. In 1821, in Bagdad, Arabia, and in 1822, in Persia and Syria. In 1823 it broke out in Antioch, Tripoli, and all along the Mediterranean coast. In 1823 and 1827, the disease continued its ravages in China and India—In 1828 the Russian Empire was again invaded. The cold of the preceding winter stayed its progress, but the summer of 1829 found it committing terrible havoc in the same localities. In 1830, it appeared in the Georgian cities, and in Poland. In 1831, Warsaw, St. Petersburg, and all the principal cities of Russia, Prussia, Austria and Italy; and the same year made its appearance in England, Scotland and Ireland. In 1832, in the month of March, it appeared in Dublin, and in May, in Paris.

Its first appearance in the New World, was in June, 1832, at Montreal and Quebec, and during its prevalence the mortality was great. Since its appearance on our shores it has been almost periodical, visiting a place in its epidemic form once in sixteen or seventeen years, and remaining from one to three years during the hot months of the year, if it found a soil congenial for its germs to propogate in. Epidemic cholera has appeared in St. Louis twice at an interval of about sixteen years. On its appearance in 1849, there were some 65,000 inhabitants in St. Louis. It is thought some 15,000 left the city; the statistics give the mortality at 6,000.

Let us compare the mortality of St. Louis with Boston and Baltimore, where strict sanitary laws were enforced in the same years. Boston appropriated a sum of money sufficient to cleanse and purify the streets and alleys and yards. The work was done thoroughly and effectively.

In St. Louis no attempt was made to remove the filth, or any way to improve the sanitary condition of the city, except what may have been done by a few individuals on their own premises.

Now compare the results; Boston, out of a population of 140,000, lost but 327 by cholera, from all causes, 5,000—while in St. Louis, with less than 65,000 inhabitants, 6,000 persons died of cholera alone.

In Baltimore $40,000 was appropriated by the city council, for the work of purification in anticipation of the coming epidemic. The result was, that out of a population of 160,000, but 853 died of cholera. Showing, I think, conclusively, that cholera can be prevented from appearing in an epidemic form. * * *

How to prevent epidemic cholera? In reference to this subject the first thing to be considered is the fact that cholera is dependent primarily on atmospheric conditions, and proximately on local conditions, impure air, being the most conspicuous. The mysterious principle, whatever it may be, generated in the great crucible of nature—whether in the jungles of India, the simoons of Arabia, or the craters of Vesuvius—may be carried on the wings of the wind over the surface of the earth, without producing more than a slight disturbance of the functions of animal life, manifested in a tendency to disease of the bowels, and general depression of the vital powers. Another poisonous principle is generated in the laboratories of terrestrial filth. Every city and town sends up its deleterious gases, its disease-laden malaria which combines with the poisonous principle with which the atmosphere is already impregnated, and thus a compound is generated, which may be the fatal cholera-poison.

Predisposition and susceptibility favor the operation of the prevailing cause, therefore sanitary measures should be promptly instituted for the thorough purification of this gas-generating foci all around us in full blast; from the offal in vacant lots; from the alleys reeking without pollution; from the gutters and back yards of tenement houses.

This is where the sanitary labors should begin. Competent sanitarians in every community, great or small, should inspect this work.

Timely and thorough *cleansing* and *disinfecting* is what "stamp out" Asiatic or sporadic cholera, better than any other means yet known to civilization.—*St. Louis Med. Journal.*—*The Clinic.*

